# Supplementary material for: Smooth Interpolating Curves with Local Control and Monotone Alternating Curvature
Source: Comput Graph Forum. 2022 Oct 6;41(5):25–38. doi: 10.1111/cgf.14600 (PMC9827861; doi:10.1111/cgf.14600)
Supplement: Supplementary file 1 — Supplement Material [file CGF-41-25-s001.zip › Local-Smooth-Interpolating-MonoCurvature/extern/clothoids/docs/api-matlab/program_listing_file_Triangle2D.m.html]

Program Listing for File Triangle2D.m — Clothoids v2.0.9

### Navigation

- index
- toc
- Clothoids »
- Program Listing for File Triangle2D.m

# Program Listing for File Triangle2D.m¶

↰ Return to documentation for file (`Triangle2D.m`)

```
classdef Triangle2D < handle
  %> MATLAB class wrapper for the underlying C++ class
  properties (SetAccess = private, Hidden = true)
    objectHandle; % Handle to the underlying C++ class instance
  end

  methods
    %>
    %> Create a new C++ class instance for the triangle object
    %>
    %> **Usage:**
    %>
    %> \rst
    %> .. code-block:: matlab
    %>
    %>    ref = Triangle2D();
    %>    ref = Triangle2D( x0, y0, x1, y1, x2, y2 );
    %>    ref = Triangle2D( p0, p1, p2 );
    %>
    %> \endrst
    %>
    %> **On output:**
    %>
    %> - `ref`: reference handle to the object instance
    %>
    function self = Triangle2D( varargin )
      self.objectHandle = Triangle2DMexWrapper( 'new', varargin{:} );
    end
    % - - - - - - - - - - - - - - - - - - - - - - - - - - - - - - - - - - -
    %> Destroy the C++ class instance
    function delete( self )
      Triangle2DMexWrapper( 'delete', self.objectHandle );
    end
    % - - - - - - - - - - - - - - - - - - - - - - - - - - - - - - - - - - -
    function obj = obj_handle( self )
      obj = self.objectHandle;
    end
    % - - - - - - - - - - - - - - - - - - - - - - - - - - - - - - - - - - -
    function str = is_type( ~ )
      str = 'Triangle2D';
    end
    % - - - - - - - - - - - - - - - - - - - - - - - - - - - - - - - - - - -
    function build( self, varargin )
      Triangle2DMexWrapper( 'build', self.objectHandle, varargin{:} );
    end
    % - - - - - - - - - - - - - - - - - - - - - - - - - - - - - - - - - - -
    %> move the triangle by `(tx,ty)`
    function translate( self, tx, ty )
      Triangle2DMexWrapper( 'translate', self.objectHandle, tx, ty );
    end
    % - - - - - - - - - - - - - - - - - - - - - - - - - - - - - - - - - - -
    %> scale triangle by `sc` factor
    function scale( self, sc )
      Triangle2DMexWrapper( 'scale', self.objectHandle, sc );
    end
    % - - - - - - - - - - - - - - - - - - - - - - - - - - - - - - - - - - -
    function [p1,p2,p3] = points( self )
      [p1,p2,p3] = Triangle2DMexWrapper( 'points', self.objectHandle );
    end
    % - - - - - - - - - - - - - - - - - - - - - - - - - - - - - - - - - - -
    %> Rotate the triangle by angle with center of rotation `(cx,cy)`
    %>
    %> **Usage:**
    %>
    %> \rst
    %> .. code-block:: matlab
    %>
    %>    ref.rotate(angle, cx, cy)
    %>
    %> \endrst
    %>
    %> **On input:**
    %>
    %> - `angle`: the angle of rotation
    %> - `cx`, `cy`: coordinates of the centre of rotation
    %>
    function rotate( self, angle, cx, cy )
      Triangle2DMexWrapper('rotate', self.objectHandle, angle, cx, cy );
    end
    % - - - - - - - - - - - - - - - - - - - - - - - - - - - - - - - - - - -
    function icode = isInside( self, x, y )
      icode = Triangle2DMexWrapper( 'isInside', self.objectHandle, x, y );
    end
    % - - - - - - - - - - - - - - - - - - - - - - - - - - - - - - - - - - -
    function dst = distanceMin( self, x, y )
      dst = Triangle2DMexWrapper( 'distanceMin', self.objectHandle, x, y );
    end
    % - - - - - - - - - - - - - - - - - - - - - - - - - - - - - - - - - - -
    function dst = distanceMax( self, x, y )
      dst = Triangle2DMexWrapper( 'distanceMax', self.objectHandle, x, y );
    end
    % - - - - - - - - - - - - - - - - - - - - - - - - - - - - - - - - - - -
    function info( self )
      Triangle2DMexWrapper( 'info', self.objectHandle );
    end
    % - - - - - - - - - - - - - - - - - - - - - - - - - - - - - - - - - - -
    function yesno = overlap( self, obj )
      yesno = Triangle2DMexWrapper( 'overlap', self.objectHandle, obj.obj_handle() );
    end
    % - - - - - - - - - - - - - - - - - - - - - - - - - - - - - - - - - - -
    function plot( self, color, varargin )
      [p1,p2,p3] = self.points();
      x = [p1(1),p2(1),p3(1),p1(1)];
      y = [p1(2),p2(2),p3(2),p1(2)];
      fill( x, y, color );
      plot( x, y, varargin{:} );
    end
    % - - - - - - - - - - - - - - - - - - - - - - - - - - - - - - - - - - -
    function plot2( self, varargin )
      [p1,p2,p3] = self.points();
      x = [p1(1),p2(1),p3(1),p1(1)];
      y = [p1(2),p2(2),p3(2),p1(2)];
      fill( x, y, varargin{:} );
    end
  end
end
```

### Quick search

### Table of Contents

- Matlab Interface Manual
- C++ API
- MATLAB API

«
hide menu

menu
sidebar
»

### Navigation

- index
- toc
- Clothoids »
- Program Listing for File Triangle2D.m

© Copyright 2021, Enrico Bertolazzi and Marco Frego.
Created using Sphinx 4.2.0.
